# Public Views on Food Addiction and Obesity: Implications for Policy and Treatment

**DOI:** 10.1371/journal.pone.0074836

**Published:** 2013-09-25

**Authors:** Natalia M. Lee, Jayne Lucke, Wayne D. Hall, Carla Meurk, Frances M. Boyle, Adrian Carter

**Affiliations:** 1 The University of Queensland, School of Population Health, Brisbane, Queensland, Australia; 2 The University of Queensland, University of Queensland Centre for Clinical Research, Brisbane, Queensland, Australia; 3 The University of Queensland, Queensland Brain Institute, Brisbane, Queensland, Australia; University of Missouri-Kansas City, United States of America

## Abstract

**Background:**

According to their advocates, neurobiological explanations of overeating, or “food addiction”, have the potential to impact public understanding and treatment of obesity. In this study, we examine the public’s acceptance of the concept of food addiction as an explanation of overeating and assess its effects upon their attitudes toward obese persons and the treatment of obesity.

**Methods and Findings:**

We conducted an online survey of 479 adults from the US (n = 215) and Australia (n = 264). There was substantial support for the idea of food addiction, particularly among obese participants. Over half favoured treating obesity as a type of addiction. Psychotherapy was believed to be the most effective treatment and educational and support programs were the preferred policies to address food addiction. There was very little support for increasing taxes on obesogenic foods. Despite the strong support for seeing obesity as a form of addiction, respondents still saw obesity as primarily the result of personal choices and emphasized the need for individuals to take responsibility for their eating.

**Conclusions:**

Our sample of the general public strongly supported the idea of obesity as a form of food addiction; but this did not translate into support of clinical and public health policies that experts believe are most likely to reduce the prevalence of obesity. The reasons for the apparent disjunction between support for food addiction and a strong emphasis on personal choice for weight warrant further examination.

## Introduction

Over a third of the global population is overweight or obese, and this percentage is increasing [1]. The populations of North America and Australia possess the highest body mass index (BMI) among developed countries [1]. Understanding the cause and spread of obesity has been the subject of much epidemiological and public health research. Recently, advances in neuroscience have suggested that addiction to specific foods may partially explain the unprecedented rates of obesity [2]–[5].

The understanding of overeating as a potentially addictive disorder, commonly termed *food addiction*, is based on animal and human neuroimaging studies [6]–[8]. These studies show that highly palatable foods, those high in sugar, fat, and/or salt, have similar effects on the brain to addictive drugs [6]. Drug abusers, compulsive eaters, and obese individuals display similar reductions in dopamine activity, poor inhibitory control and reduced sensitivity to pleasure [6], [9]. Patterns of eating in some individuals also closely resemble the behaviour of drug-addicted individuals, i.e. they show tolerance, withdrawal, craving, and cross sensitization [6]. Many compulsive eaters and obese individuals, particularly those with binge eating disorder, satisfy the DSM-IV criteria for substance dependence when these are applied to the consumption of specific foods [10], [11]. It was suggested that overeating be classified as an addictive disorder in the 5^th^ revision of the DSM [12], [13].

Prominent neuroscientists argue that neurobiological explanations of overeating may improve treatment and encourage greater public support for public health policies to reduce overeating [3], [7], such as increased taxation and regulation of processed foods unnaturally high in sugar and fat, as has happened in Hungary, Denmark, and New York City, USA [6]. A number of studies have examined the strength of the evidence for the concept of food addiction [4], , and its perceived impact upon treatment and policy [6], [7], but the impact of an addiction model of obesity on public opinion has not been tested.

What policies would the public support if its members accepted that overeating was a type of addictive disorder? How would this new understanding affect public views and attitudes towards obese persons? How would this view affect individuals’ eating and their efforts to overcome obesity?

The present study examined public support for an addiction model of obesity, and the possible impacts of neurobiological explanations of overeating and obesity on public attitudes toward obese persons and support for different policies to treat and prevent obesity. Participants in the US and Australia were surveyed online to identify any cross-cultural differences between public attitudes in two developed Westernized countries that have high rates of obesity.

## Methods and Procedures

### Ethics Statement

This study was approved by a Health Research Ethics Committee at The University of Queensland and complied with the ethical standards laid down in the 1964 Declaration of Helsinki. Before commencing the study, participants read and indicated their acceptance of an informed consent statement. Participants were informed about their right to conclude the experiment at any time. All participants’ data were analyzed and reported anonymously.

### Data Source

This study was conducted from July to August 2012 using a sample of US and Australian residents 18 years and older recruited through online advertising by Google AdWords and Facebook. Supplementary recruitment included an online staff newsletter at The University of Queensland and snowball sampling. A small financial incentive to participate was provided in the form of an opportunity to win a $50 gift card.

### Survey Measures

The online survey involved a series of multiple-choice items and 5-point Likert scales to assess public attitudes toward obesity, views on the causes and risk factors for obesity, and degree of endorsement of the concept of food addiction. The survey instrument was initially piloted on 20 individuals and the questions revised to improve comprehension (see supplementary material).

#### Understanding of obesity

Participants were asked three questions to assess their understanding of the causes of obesity and its risk factors. The first question was a multiple-choice question to assess what participants believed to be the *main* cause of obesity. “Biological causes” and “genetics or family history” were combined during analysis as the two represent causes external to personal control (see Table S1).

Participants’ attitudes toward obesity were assessed by asking them to answer a series of questions in response to the following vignette:

“Sarah is 5′3″ (161 cm) and weighs 200 pounds (91 kg) at 30 years of age. She has tried, unsuccessfully, to lose weight on multiple occasions. Doctors have told Sarah that she is obese and have expressed concerns about her health.”

The questions were adapted from two validated social surveys of mental health stigma [14], [15] to measure weight-based stigma. The four questions that measured perceived control and responsibility for weight and weight gain (as determined by the vignette) were based on a 5-point Likert scale, and included, “How much control does Sarah have over her eating” and “How responsible is Sarah for losing weight” (see Table S2).

Participants were asked three questions to explore their understanding of current treatments for obesity, namely, what they thought were: the most common, and the most and least effective treatments for obesity (see Table S3).

#### Endorsement of food addiction

Participants were asked to indicate their level of agreement with seven statements on the addictive properties of food, and the relationship between drugs and obesity (see Table S4). Five statements were used to calculate an overall measure of their support for food addiction (the *food addiction support index* or FASI). These were: (i) Some foods (particularly those high in sugar or fat) are addictive; (ii) Some foods can be as addictive as drugs (e.g. alcohol, tobacco, cocaine); (iii) Sugar is addictive; (iv) Certain styles of eating (e.g. overeating, compulsive eating, binge eating) are similar to addiction; and (v) Obesity should be treated as an addiction. An index range of 0 to 20 was calculated by adding the scores of each of the five possible response options (e.g. ‘Strongly disagree’ = 0, ‘Disagree’ = 1, ‘Don’t know’ = 2, ‘Agree’ = 3 and ‘Strongly agree’ = 4). Scores on the food addiction support index were divided into 3 categories: no support (0–7), ambivalence (8–12), and high support (13–20). The FASI was then used to examine the relationship between food addiction support and views on the treatment and policy options for reducing obesity and overeating.

Participants were provided with the following statement about recent neuroscientific research on overeating and obesity:

“Recent scientific research suggests that high sugar or high fat foods can produce changes in the brain similar to addictive drugs and that these foods may become addictive to some individuals.”

After reading the statement, participants were asked about their prior awareness of the neuroscience of overeating and their agreement with it (see Table S5).

#### Demographic, weight and eating information

Demographic details, family and personal history of addictive behaviours, related psychiatric conditions and obesity, and participants’ self-reported height and weight (to calculate BMI) were collected.

### Statistical Analysis

Means and standard deviations were computed for the continuous variables and standardized proportions for all variables of interest. Chi-square and logistic regression analyses were calculated using the statistical software R (R Foundation for Statistical Computing, Vienna, Austria). For the Chi-square analyses, Likert responses were reduced to a dichotomous response, ‘Agree’ or ‘Disagree’ (and ‘Don’t know’ where applicable), for the simplification of results and to maximise contrast in responses.

## Results

### Sample Characteristics

A total of 610 individuals began the online survey, with 79% completing the study without error (n = 479), yielding 215 US and 264 Australian participants. There were no significant differences in gender, median age, race, or education based upon country of residence (see Table 1). Compared with the nationwide averages in both the US and Australia, our samples are better educated, contain a lower proportion of minorities, and are predominately female.

**Table 1:** Demographic characteristics of sample.

Sample Characteristics	Total (n = 479)n (%)	US (n = 215)n (%)	Australia (n = 264)n (%)
**Sex**			
*Female*	383 (80)	170 (79)	213 (81)
*Male*	93 (19)	43 (20)	50 (19)
**Age**			
*18–24*	73 (15)	32 (15)	41 (16)
*25–34*	154 (32)	55 (26)	99 (38)
*35–44*	87 (18)	42 (20)	45 (17)
*45–54*	82 (17)	35 (16)	47 (18)
*55–64*	59 (12)	40 (19)	19 (7)
*65–84*	24 (5)	11 (5)	13 (5)
**Self-reported BMI**			
*Underweight, BMI <18.5 kg/m^2^*	14 (3)	5 (2)	9 (3)
*Normal weight, BMI 18.5–24.9 kg/m^2^*	228 (48)	93 (43)	135 (51)
*Overweight, BMI 25–29.9 kg/m^2^*	104 (22)	46 (21)	58 (22)
*Obesity, BMI >30 kg/m^2^*	133 (28)	71 (33)	62 (23)
**Education**			
*High school or GED completed*	75 (16)	32 (15)	43 (16)
*2-Year vocational or technical degree*	33 (7)	22 (10)	11 (4)
*College graduate*	166 (35)	80 (37)	86 (33)
*Postgraduate degree*	204 (43)	80 (37)	124 (47)
**Household Income (USD)**			
*<$25,000*	51 (11)	32 (15)	19 (7)
*$25,000–49,999*	86 (18)	44 (20)	42 (16)
*$50,000–74,999*	84 (18)	37 (17)	47 (18)
*$75,000–99,999*	71 (15)	30 (14)	41 (16)
*$100,000+*	187 (39)	72 (33)	115 (44)

Both samples reflected the prevalence of obesity and median age in their respective countries [16]–[18] but differed from their respective national populations in gender, age, race, education, income, and BMI. Both the US and Australian samples contained a larger proportion of normal weight participants, a lower proportion of overweight participants, and were similar in the proportion of obese participants compared with the nationwide averages of each respective country [16], [18]. Our sample was slightly lighter than the general population in both the US and Australia. Stratification based on BMI [19], [20] showed 48% of the total sample was of normal weight (BMI 18.5–24.9), 22% were overweight (BMI 25.0–29.9), and 28% were obese (BMI of 30+). The mean BMI of participants was 27.4 (SD = 8.2).

### Understanding of Obesity and Food Addiction

**Figure 1:**
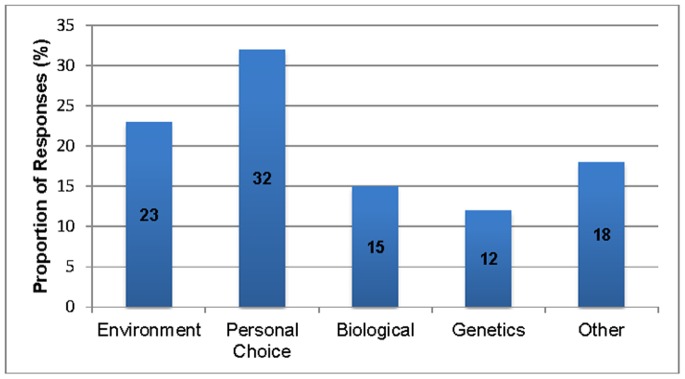
Responses as to the main cause of obesity.

One third of participants said personal choice (32%) was the main cause of obesity, 27% ascribed it either to biological and genetic causes, and 23% chose the environment. A sizeable minority (18%) chose “other”, with most of these participants indicating that obesity was caused by a combination of factors (see Figure 1).

Over 90% of participants attributed obesity to overeating. This was higher than the proportion (72%) in a recent systematic review [21]. The majority of participants (57%) agreed that there is a medical cause to obesity, although over a quarter were unsure. Views on the causes of obesity did not differ significantly by country of residence.

Almost three quarters (72%) of participants believed that an addiction to certain foods caused obesity. Just over half (54%) agreed that obesity should be treated as an addiction, and 64% were prepared to classify obesity as an eating disorder. Most (86%) participants thought that certain foods are addictive (79% in the case of sugar) and 80% believed that some foods could be as addictive as alcohol, nicotine and cocaine. US and Australian participants did not differ in the proportions who agreed that obesity was caused by a food addiction (69% v. 74%) or who considered obesity to be an eating disorder (60% v. 67%). A significantly lower proportion of US (73%) than Australian (86%) participants agreed that obesity was harmful to society (OR = 0.49, 95% CI 0.29–0.84), and that obesity should be treated as an addiction: 47% US vs. 59% Australia (OR = 0.59, 95% CI 0.38–0.92) (see Tables S6a and b).

Two thirds (69%) of participants were aware of research suggesting that foods could be addictive in the sense of producing changes in the brain similar to drugs of abuse. 81% of all participants supported this view. Participants from the US and Australia did not differ in their awareness and acceptance of neuroscientific evidence for food addiction.

### Control and Responsibility Over Food Consumption and Weight

Three quarters (76%) of participants agreed that Sarah was responsible for losing weight and half agreed that she was responsible for becoming obese. Participants were strongly divided as to whether Sarah exhibited control over her weight (see Table 2).

**Table 2:** Responses to control and responsibility based on vignette.

How much control does Sarah have over her…^1^	Control n (%)	No control n (%)
weight?	193 (40)	233 (49)
eating?	263 (55)	171 (36)
**How responsible is Sarah for…** 1	**Responsible n (%)**	**Not responsible n (%)**
becoming obese?	238 (50)	181 (38)
losing weight?	365 (76)	87 (18)

1 Proportions listed do not include ambiguous (‘don’t know’) responses.

Responses to questions on control and responsibility based on the vignette differed between country of residence: a significantly larger proportion of Australians (55%) than Americans (43%) viewed Sarah as responsible for becoming obese (X^2^(1) = 5.644, p<0.05). There were no significant differences on the additional measures of control and responsibility by country of residence.

### Impact on the Treatment of Obesity

**Figure 2:**
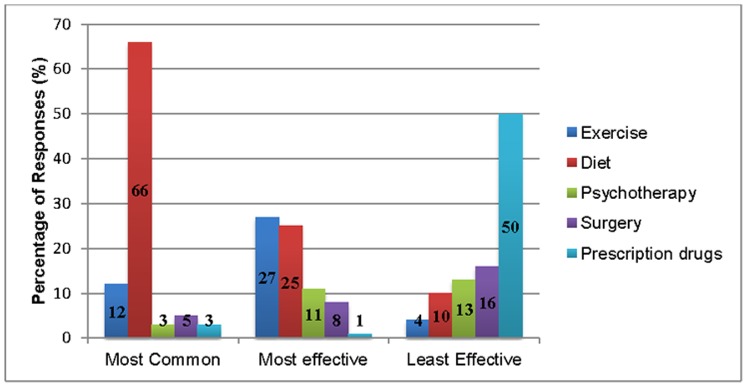
Proportion of responses based on most common, most effective, and least effective treatments of obesity.

Participants’ views on the most commonly used and the most and least effective treatments of obesity are given in Figure 2. Two-thirds believed that diet was the most common treatment of obesity but only one quarter believed it to be the most *effective*. Just over a quarter of participants (27%) thought that exercise was the most effective treatment of obesity. Half of the participants thought that prescription drugs were the least effective treatment of obesity, followed by surgery (16%). Participants’ responses varied only slightly by country of residence: 31% of US participants believed that exercise was most effective whereas 30% of Australians thought that diet was most effective.

Psychotherapy or counseling was listed by 44% of participants as the most effective treatment for a food addiction, followed by dietary changes (22%). Educational and support groups were thought by 33% to be the most effective policy to address food addiction. Restrictions on advertising had the least support (5%). Over half of the participants (57%) disagreed that imposing a tax on certain foods would lower rates of obesity and 49% did not think that such a tax would be helpful to society. There were no significant differences between US and Australian participants on the most effective treatment and policy changes needed to reduce an addiction to certain foods. While the participants were aware of and supported the concept of food addiction, this did not change their attitudes toward obese individuals or the most effective method of treating obesity in 75% and 53% of participants, respectively.

#### Food addiction support index

Support for food addiction did not influence participants’ endorsement of the most effective policy to address food addiction (see Table S7). Support for the taxation of foods also did not vary with support for food addiction (see Table S8). When examining the impact of food addiction support on measures of control and responsibility based on the vignette, participants in the high support group were over twice as likely (OR = 2.26, 95% CI 1.02–4.80) as participants with no support to hold Sarah responsible for losing weight (see Tables 3 and 4). There was no significant difference between each of the categories regarding Sarah’s perceived control for her weight and eating, as well as for her perceived responsibility for becoming obese. The demographic and BMI data for each FASI category are provided in Table S9.

**Table 3:** Impact of food addiction support and BMI on control based on responses to the vignette.

Sarah has controlover her …	Weight		Eating	
Agreement	OR	95% CI	OR	95% CI
**FASI**				
No Support	Reference		Reference	
Ambivalence	1.159	0.499–2.719	0.691	0.278–1.652
High Support	1.067	0.520–2.228	0.546	0.248–1.130
**BMI**				
Normal	Reference		Reference	
Overweight	0.852	0.522–1.387	0.809	0.490–1.343
Obese	0.355***	0.213–0.581	0.433	0.268–0.696

*** p<0.001.

FASI = Food addiction support index: No support (0–7); Ambivalence (8–12) and High support (13–20).

BMI = Body mass index: Normal weight 18.5–24.9; Overweight 25–29.9; Obese >30.

**Table 4:** Impact of food addiction support and BMI on responsibility based on responses to the vignette.

Sarah isresponsible for …	Becoming obese		Losing weight	
Agreement	OR	95% CI	OR	95% CI
**FASI**				
No Support	Reference		Reference	
Ambivalence	1.802	0.772–4.287	2.086	0.786–5.584
High Support	2.003	0.981–4.176	2.259*	1.020–4.803
**BMI**				
Normal	Reference		Reference	
Overweight	0.842	0.512–1.423	1.011	0.516–2.067
Obese	0.341***	0.209–0.553	0.316***	0.180–0.551

* p<0.05,

*** p<0.001.

FASI = Food addiction support index: No support (0–7); Ambivalence (8–12) and High support (13–20).

BMI = Body mass index: Normal weight 18.5–24.9; Overweight 25–29.9; Obese >30.

#### Body mass index

Views on the causes of obesity varied with BMI (X^2^(4) = 33.963, p<0.001). Biological and genetic causes were more often endorsed by obese participants (M = 31.2, SD = 9.8), environmental causes by overweight participants (M = 26.2, SD = 6.1), and personal choice by normal weight participants (M = 24.9, SD = 6.1). Obese participants were less likely to state that overeating causes obesity than normal weight participants (OR = 0.29, 95% CI 0.13–0.64). Support for the view that certain foods could be as addictive as drugs increased with BMI.

Obese participants were less than a third as likely as their normal and overweight counterparts to view obesity as harmful to society (OR = 0.30, 95% CI 0.16–0.54) (see Tables S6a and S6b). Obese participants were also less supportive of imposing a tax on foods than normal and overweight participants (OR = 0.52, 95% CI 0.21–0.58) (see Table S8).

Participants’ awareness of certain foods’ addictive potential and their agreement with this did not significantly differ by BMI. Obese individuals were twice as likely to report a change in their views about obese individuals (p<0.05) and obesity treatment (p<0.001) after hearing about neuroscientific explanations of addiction than were normal weight participants.

Obese individuals believed that Sarah had less control over her eating and weight (OR = 0.36, 95% CI 0.21–0.58) and was less responsible for becoming obese (OR = 0.34, 95% CI 0.21–0.55) and losing weight (OR = 0.32, 95% CI 0.18–0.55) than normal and overweight participants (see Tables 3 and 4). Perceived personal responsibility for weight decreased as BMI increased.

## Discussion

We have speculated previously that an addiction model of obesity could focus attention on the medical causes and treatments of obesity (commonly referred to as medicalization) at the expense of broader public health approaches [6]. Similar concerns have been made about the medicalization of other addictions [22], [23]. Medical treatments include pharmacotherapies (e.g. Belviq, Qsymia, and novel concomitant therapies) [6], , gastric surgery (e.g. laparoscopic gastric bypass, adjustable gastric band, laparoscopic sleeve gastrectomy, and biliopancreatic diversion) [25], and even neurosurgery (e.g. deep brain stimulation) [26], [27]. An addiction model of obesity could also be seen to undermine individuals’ belief in their ability to control their weight and absolve them of responsibility for overcoming their condition [28]. Similar concerns have been raised about neurobiological explanations of drug addiction [29].

Our findings provided little support for this view. While we found strong public support for the view that certain foods are addictive (86% of those surveyed) and that food addiction may partly explain some cases of obesity, this did not translate into acceptance of a medicalised approach to treating obesity.

For example, 79% of participants believed that sugar in particular was addictive, almost three-quarters attributed obesity to an addiction to certain foods, and 75% believed that these foods were as addictive as alcohol and cocaine. Despite this strong endorsement of the food addiction model of obesity, the majority of our sample believed that obese individuals retained control over their eating (55% ascribed them as having control v. 36% having no control). Half of all participants also thought that Sarah was responsible for becoming obese while over three-quarters (76%) thought that she was responsible for losing weight. Perceived responsibility for losing weight was particularly high among those participants who strongly supported the food addiction view of overeating. A loss of control over use is a defining feature of addiction that is included in the major diagnostic systems (ICD-10 and DSM-IV). This loss of control is often seen as entailing a reduced responsibility for both becoming addicted and for overcoming addiction. Support for the food addiction model of obesity, however, did not remove an individual’s ability to control their eating or their responsibility for losing weight. This may explain why very few participants (9.8%) supported forcing Sarah into weight loss treatment. Obesity was still viewed as a condition that individuals had to overcome through personal choice and will power.

Approximately half of participants believed that the obese individual in the vignette had no control over their *weight*. Participants were more willing to hold that obese individuals had less control over their weight than their eating. This suggests that a failure to lose weight was not seen as simply the result of an inability to control eating, but may be explained by other factors that could include a medical condition, genetic differences or a lack of exercise. Overeating was still considered an important factor, with over 90% of our participants stating that obesity was due to overeating.

The importance of personal choice in overeating and obesity was reflected in participants’ understandings of the causes of obesity. A third believed that personal choice was the main cause of obesity, while 27% believed that biology (including genetic factors) was the primary cause. This is consistent with the commonly held view among the general US population that obese individuals are personally responsible for their weight [7]. More respondents attributed obesity to biological or genetic causes in the vignette, however. Vignettes measure participant’s reactions to specific examples as opposed to eliciting more general responses [14]. It is important to note that our use of a female character in the vignette could have elicited different responses than a male may have as other research suggests that males and females experience weight-based stigma differently [21], [30].

### Implications for Treatment and Policy

Our findings indicate that while participants were willing to accept that some foods can be addictive, this did not entail support for medical treatments of obesity or change the strong emphasis placed on obese persons’ responsibility for their weight.

There are several possible explanations for this discrepancy. It may reflect the fact that the public places a high emphasis on the role of personal responsibility and choice in overcoming obesity, as indicated above. It may also reflect the view that medical treatments of obesity are of limited effectiveness. Very few thought that medical interventions such as prescription drugs (1%), surgery (8%) or psychotherapy (11%) would be effective. Diet was seen as the most common treatment of obesity by two-thirds of respondents but only a quarter of respondents believed it to be effective. It is not clear whether the perceived ineffectiveness of diet was attributed to a failure of individuals to adhere to dietary restrictions, or whether other factors are perceived to be more important in the treatment of obesity. Exercise was seen as an under-utilised treatment, suggesting that the public see a sedentary lifestyle as a barrier to overcoming obesity.

There was only limited public support for frequently advocated population-based approaches to reducing obesity, such as imposing taxes on highly addictive foods or banning food advertising. In fact, 57% thought that taxation would be ineffective in reducing obesity and less than half thought that it would be helpful to society. Further analyses based on the FASI were not seen to predict support for taxation. As our participants had a higher level of educational attainment than the general public, our findings may overestimate public support for taxation as a policy to reduce obesity. Our results found that support for food taxation was greatest among the more educated participants (see Table S6a). Among our sample as a whole, educational programs received the strongest support as public health policies. These findings suggest that our participants preferred approaches targeted at the individual, especially those that were elective (e.g. educational programs), than broad population-based approaches that aim to reduce weight and overconsumption in the whole population (i.e. food taxation and advertising bans).

Recent studies show greater support for food taxation if the funds raised are used to address childhood obesity [31]. This suggests an alternative way for advocates of food taxation to present their case. Policy measures for the health protection of children receive considerable public attention and support. The inclusion of neuroscience research on overeating may strengthen the argument for initiatives to prevent childhood obesity that involve restrictions on marketing and advertising of the most harmful foods.

Population-based approaches, such as taxation and regulation, have consistently shown to improve public health, such as reducing problem alcohol use and tobacco smoking [3]. Proposals to adopt similar approaches to the taxation and regulation of foodstuffs are often met with public opposition [32]. The strongest *opposition* comes from the food industry itself [33] that disavows responsibility for creating obesogenic environments and stress individual responsibility for eating. The public seem to be receptive to these arguments. Advocates of public health policies for obesity should anticipate resistance to policy measures that are seen to ignore obese individuals’ responsibility for overeating and excess weight.

### Attitudes by Country of Residence

On the whole, the US and Australian samples shared similar views on their understandings of obesity and their preparedness to endorse the concept of food addiction. There were several notable differences that may warrant further examination. Australian participants were more aware of the harmful effects of obesity on society. A significantly larger proportion of Australians thought that obesity should be treated as an addiction. This was unexpected given that the majority of the research on food addiction has been done in the US. Australian participants were also more likely to hold obese individuals responsible for becoming obese. There was significantly less support for taxation of foods in the US, possibly reflecting a greater reluctance to use taxes to benefit health or influence behaviour in America. These discrepancies could also be due to differences in how participants were recruited in the two countries, such as the supplementary recruitment of Australian participants through an online staff newsletter at The University of Queensland.

### Attitudes and Individual Obesity

Our study found that obese individuals were significantly more supportive of the concept of food addiction than their overweight and normal weight counterparts. They were also more supportive of treating obesity as a type of food addiction. Obese participants were more likely than non-obese participants to believe that obese individuals have less control and responsibility over their eating and weight. This may also explain the lower level of support for food taxation among obese participants. Heavy smokers are similarly less supportive of tobacco taxation policies than are non-smokers [34]. A diagnosis of food addiction may also reduce some of the guilt and self-blame that obese individuals feel in response to their eating and weight. In accordance with this view, obese participants showed more support for external causes of obesity and were less likely to agree that obesity was caused by overeating. These findings may have significant implications for the clinical treatment of obesity and the communication of food addiction models of addiction. Emphasising food addiction may further undermine obese individuals’ perceived control over their eating and self-efficacy. Further research is urgently needed to examine the impact of food addiction understandings on obesity and obese individuals.

### Limitations and Future Directions

Our participants were not representative of the general public in either the US or Australia. Female respondents were over-represented, possibly reflecting a greater interest among women in weight-based issues. A large proportion of participants (43%) also had a postgraduate qualification, potentially increasing their awareness of and preparedness to accept findings from neuroscientific research. Future research should be done on more representative samples of the population in both the US and Australia. The vignette used in this study only elicited responses directed towards obese women. Future studies should also examine attitudes towards obese males, and possibly individuals from different ethnicities, social strata or age groups. We were also unable to measure the association between FASI and BMI with the current study design. Future research should be conducted with this specific aim and should examine the impact of these associations on treatment and policy responses to obesity.

## Conclusion

We found that apparently strong public acceptance of neurobiological explanations of overeating and obesity can co-exist with the view that personal choice is the predominant cause of obesity. In our sample, obese participants were more likely to support the view that obesity represents an addiction to certain foods. The apparent failure of neurobiological explanations of overeating and obesity to alter public views toward obese individuals and the treatment of obesity suggests that these explanations have not yet had the beneficial impacts assumed by their advocates. As the concept of food addiction is developed, its advocates need to pay greater attention to its effects on stigma, treatment and policy and to assessing whether its net impact on public health is likely to be harmful or beneficial.

## Supporting Information

Table S1 Questions used to measure causality and risk factors for obesity.
(DOCX)

Table S2 Questions used to measure views on stigma, and control and responsibility.
(DOCX)

Table S3 Questions used to measure the perception of treatments for obesity.
(DOCX)

Table S4 Questions used to assess participants’ understanding of food addiction.
(DOCX)

Table S5 Questions used to assess awareness, agreement, and impact of food addiction on obesity.
(DOCX)

Table S6 (a). Percentage agreement based on sociodemographic characteristics in relation to addictive foods and obesity treatment. (b). Factors influencing support for obesity and food addiction treatment.
(DOCX)

Table S7 Endorsement of policy responses based upon level of support addiction support.
(DOCX)

Table S8 Impact of food addiction support and BMI on support for food taxation.
(DOCX)

Table S9 Demographic characteristics of sample based upon Food Addiction Support Index (FASI).
(DOCX)
