# Supplementary material for: Public Views on Food Addiction and Obesity: Implications for Policy and Treatment
Source: PLoS One. 2013 Sep 25;8(9):e74836. doi: 10.1371/journal.pone.0074836 (PMC3783484; doi:10.1371/journal.pone.0074836)
Supplement: Table S1 — Questions used to measure causality and risk factors for obesity. (DOCX) [file pone.0074836.s001.docx]

Table S1. Questions used to measure causality and risk factors for obesity.

| **Causality and Risk Factors** |
| --- |
| What do you think is the main cause of obesity? |
| *[Biological causes/Environment/Genetics or Family history/Personal choice/Other]* |
| Do you think there is a medical cause of obesity? |
| *[Yes/No/Unsure]* |
| To what extent do you agree with the following statements? |
| *[Strongly agree/Agree/Disagree/Strongly disagree/Don't know]* |
| Obesity is caused by overeating. |
| Obesity is caused by a lack of exercise. |
| Obesity is caused by an addiction to certain foods. |
| Obesity is considered an eating disorder. |
| Like hair loss, excess weight comes with age. |
| Most individuals overeat without becoming obese. |
| Excess weight is preventable. |
| Obesity is preventable. |
| Obesity is treatable. |
| Obesity is a risk factor for cancer. |
| Obesity is a risk factor for cardiovascular disease. |
| Obesity is a risk factor for depression. |
| Obesity is a risk factor for complications during pregnancy. |
| Obesity is a risk factor for dementia. |
| Obesity is a risk factor for premature death. |
