# Supplementary material for: Public Views on Food Addiction and Obesity: Implications for Policy and Treatment
Source: PLoS One. 2013 Sep 25;8(9):e74836. doi: 10.1371/journal.pone.0074836 (PMC3783484; doi:10.1371/journal.pone.0074836)
Supplement: Table S2 — Questions used to measure views on stigma, and control and responsibility. (DOCX) [file pone.0074836.s002.docx]

Table S2. Questions used to measure views on stigma, control and responsibility for weight (gain).

| **Stigmatisation and Discrimination** |
| --- |
| I would be comfortable if Sarah was my colleague at work. |
| I would be comfortable inviting Sarah to a dinner party. |
| I would be comfortable having Sarah as an in-law. |
| *[Strongly agree/Agree/Disagree/Strongly disagree/Don't know]* |
| How likely do you think it would be for Sarah's husband to leave her? |
| How likely do you think it would be for Sarah to get fired? |
| How likely do you think it would be for Sarah to gain additional weight? |
| *[Very likely/Quite likely/Unlikely/Very unlikely/Don't know]* |
| Sarah should seek treatment for an eating disorder. |
| Sarah should be forced into weight-loss treatment. |
| *[Strongly agree/Agree/Disagree/Strongly disagree/Don't know]* |
| How much control does Sarah have over her weight? |
| How much control does Sarah have over her eating? |
| *[Total control/A lot of control/Some control/No control/Don't know]* |
| How responsible is Sarah for becoming obese? |
| How responsible is Sarah for losing weight? |
| *[Totally responsible/Mostly responsible/Somewhat responsible/Not at all responsible/Don't know]* |
| What is the main cause of Sarah's obesity? |
| *[Biological causes/Environment/Genetics or Family history/Personal choice/Other]* |
