# Supplementary material for: Public Views on Food Addiction and Obesity: Implications for Policy and Treatment
Source: PLoS One. 2013 Sep 25;8(9):e74836. doi: 10.1371/journal.pone.0074836 (PMC3783484; doi:10.1371/journal.pone.0074836)
Supplement: Table S3 — Questions used to measure the perception of treatments for obesity. (DOCX) [file pone.0074836.s003.docx]

Table S3. Questions used to measure the perception of treatments for obesity.

| **Treatment Endorsement** |
| --- |
| In your opinion, what is the most common treatment of obesity? |
| In your opinion, what is the most effective treatment of obesity? |
| In your opinion, what is the least effective treatment of obesity? |
| *[Diet/Exercise/Prescription drugs/Surgery/Therapy or counseling/Other]* |
