# Supplementary material for: Public Views on Food Addiction and Obesity: Implications for Policy and Treatment
Source: PLoS One. 2013 Sep 25;8(9):e74836. doi: 10.1371/journal.pone.0074836 (PMC3783484; doi:10.1371/journal.pone.0074836)
Supplement: Table S4 — Questions used to assess participants’ understanding of food addiction. (DOCX) [file pone.0074836.s004.docx]

Table S4. Questions used to assess participants’ understanding of food addiction.

| **Impact of Food Addiction on Obesity** |
| --- |
| To what extent do you agree with the following statements? |
| *[Strongly agree/Agree/Disagree/Strongly disagree/Don't know]* |
| Some foods (particularly those high in sugar or fat) are addictive. |
| Some foods can be as addictive as drugs (e.g. alcohol, tobacco, cocaine). |
| Sugar is addictive. |
| Certain styles of eating (e.g. overeating, compulsive eating, binge eating) are similar to addiction. |
| Obesity is harmful to society in general. |
| Obesity should be treated as an addiction. |
| Treating an addiction to certain foods could decrease rates of obesity. |
| If certain foods were proven to be addictive, what would be the most effective treatment for an addiction to certain foods? |
| *[Diet/Exercise/Prescription drugs/Surgery/Therapy or counseling/Don't know/Other]* |
| What would be the most effective policy change needed to reduce an addiction to certain foods? |
| *[Educational and support programs/Health insurance coverage for treatment/Increasing access or availability of healthy foods/Limiting access or availability of problem foods/Restrictions on food advertising/Subsidies for healthy foods/Taxation of problem foods/Don't know/Other]* |
| A tax on certain foods (e.g. high fat, high sugar) would decrease rates of obesity. |
| A tax on certain foods (e.g. high fat, high sugar) is helpful to society in general. |
| *[Strongly agree/Agree/Disagree/Strongly disagree/Don't know]* |
