# Supplementary material for: Public Views on Food Addiction and Obesity: Implications for Policy and Treatment
Source: PLoS One. 2013 Sep 25;8(9):e74836. doi: 10.1371/journal.pone.0074836 (PMC3783484; doi:10.1371/journal.pone.0074836)
Supplement: Table S5 — Questions used to assess awareness, agreement, and impact of food addiction on obesity. (DOCX) [file pone.0074836.s005.docx]

Table S5. Questions used to assess awareness, agreement, and impact of food addiction on obesity.

| **Food Addiction Awareness:** Recent scientific research suggests that high sugar or high fat foods can produce changes in the brain similar to addictive drugs and that these foods may become addictive to some individuals. |
| --- |
| Were you previously aware of this? |
| *[Yes/No]* |
| To what extent do you agree with this view? |
| *[Strongly agree/Agree/Disagree/Strongly disagree/Don't know]* |
