# Supplementary material for: Public Views on Food Addiction and Obesity: Implications for Policy and Treatment
Source: PLoS One. 2013 Sep 25;8(9):e74836. doi: 10.1371/journal.pone.0074836 (PMC3783484; doi:10.1371/journal.pone.0074836)
Supplement: Table S6 — (a). Percentage agreement based on sociodemographic characteristics in relation to addictive foods and obesity treatment. (b). Factors influencing support for obesity and food addiction treatment. (DOCX) [file pone.0074836.s006.docx]

Table S6(a). Percentage agreement based on sociodemographic characteristics in relation to addictive foods and obesity treatment.

|  | Obesity is harmful to society  n (%) | Obesity should be treated as an addiction  n (%) | Treatment for food addiction could decrease obesity  n (%) | Food tax would decrease obesity  n (%) | Food tax is helpful  n (%) |
| --- | --- | --- | --- | --- | --- |
| **BMI** | | | | | |
| Normal weight | 197 (86) | 106 (46) | 133 (58) | 76 (33) | 97 (43) |
| Overweight | 88 (85) | 68 (65) | 69 (66) | 39 (38) | 49 (47) |
| Obesity | 87 (65) | 81 (61) | 89 (67) | 32 (24) | 39 (29) |
| **Education** | | | | | |
| Non-university | 75 (69) | 63 (58) | 70 (64) | 28 (26) | 28 (26) |
| College graduate | 139 (84) | 92 (55) | 112 (67) | 45 (27) | 63 (38) |
| Postgraduate | 171 (84) | 104 (51) | 115 (56) | 79 (39) | 99 (49) |
| **Country of Residence** | | | | | |
| Australia | 228 (86) | 157 (59) | 170 (64) | 94 (35) | 117 (44) |
| US | 157 (73) | 102 (47) | 127 (59) | 58 (27) | 73 (34) |

BMI = Body mass index: normal weight 18.5-24.9; overweight 25-29.9; obese > 30

Table S6(b). Factors influencing support for obesity and food addiction treatment.

| **Predicting agreement** | **Obesity is harmful to society** | | **Obesity should be treated as an addiction** | |
| --- | --- | --- | --- | --- |
|  | OR | 95% CI | OR | 95% CI |
| **BMI** |  |  |  |  |
| Normal  Overweight  Obese | Ref.  0.877  0.299*** | 0.419-1.915  0.163-0.537 | Ref.  2.221**  1.855* | 1.268-3.998  1.108-3.154 |
| **Education** |  |  |  |  |
| Non-University  College Graduate  Postgraduate | Ref.  2.360*  1.982* | 1.217-4.629  1.039-3.783 | Ref.  0.707  0.718 | 0.384-1.280  0.393-1.290 |
| **Country of Residence** |  |  |  |  |
| Australia  US | Ref.  0.493** | 0.287-0.837 | Ref.  0.591* | 0.379-0.918 |

p<0.05*, p<0.01**, p<0.001***

BMI = Body mass index: normal weight 18.5-24.9; overweight 25-29.9; obese > 30

| **Predicting agreement** | **Treatment for food addiction could decrease obesity** | | **Food tax would decrease obesity** | | **Food tax is helpful** | |
| --- | --- | --- | --- | --- | --- | --- |
|  | OR | 95% CI | OR | 95% CI | OR | 95% CI |
| **BMI** |  |  |  |  |  |  |
| Normal  Overweight  Obese | Ref.  2.062  1.136*** | 0.993-4.640  0.629-2.085 | Ref.  1.092  0.637 | 0.655-1.810  0.381-1.052 | Ref.  1.132  0.581* | 0.687-1.867  0.356-0.940 |
| **Education** |  |  |  |  |  |  |
| Non-University  College Graduate  Postgraduate | Ref.  0.877  0.645 | 0.413-1.801  0.307-1.298 | Ref.  1.049  1.786* | 0.589-1.889  1.033-3.142 | Ref.  1.649  2.525*** | 0.945-2.924  1.470-4.414 |
| **Country of Residence** |  |  |  |  |  |  |
| Australia  US | Ref.  0.504* | 0.293-0.857 | Ref.  0.718 | 0.472-1.088 | Ref.  0.701 | 0.467-10.48 |

p<0.05*, p<0.01**, p<0.001***
