# Supplementary material for: Public Views on Food Addiction and Obesity: Implications for Policy and Treatment
Source: PLoS One. 2013 Sep 25;8(9):e74836. doi: 10.1371/journal.pone.0074836 (PMC3783484; doi:10.1371/journal.pone.0074836)
Supplement: Table S7 — Endorsement of policy responses based upon level of support addiction support. (DOCX) [file pone.0074836.s007.docx]

Table S7. Endorsement of policy responses based upon level of support addiction support.

| Row percentage endorsement of n (%) based on food addiction support index | | | | | |
| --- | --- | --- | --- | --- | --- |
|  | Educational and Support Programs | Health Insurance Coverage | Access and Availability of Foods | Restrictions on Food Advertising | Subsidies and Taxation |
| **Food Addiction Support Index** | | | | | |
| No Support | 9 (29) | 4 (13) | 9 (29) | 1 (3) | 8 (26) |
| Ambivalence | 29 (45) | 7 (11) | 14 (22) | 2 (3) | 13 (20) |
| High Support | 119 (37) | 36 (11) | 99 (31) | 22 (7) | 47 (15) |

FASI = Food addiction support index: No support (0-7); Ambivalence (8-12) and High support (13-20)
