# Supplementary material for: Public Views on Food Addiction and Obesity: Implications for Policy and Treatment
Source: PLoS One. 2013 Sep 25;8(9):e74836. doi: 10.1371/journal.pone.0074836 (PMC3783484; doi:10.1371/journal.pone.0074836)
Supplement: Table S8 — Impact of food addiction support and BMI on support for food taxation. (DOCX) [file pone.0074836.s008.docx]

Table S8. Impact of food addiction support and BMI on support for food taxation.

| **Prediction of agreement** | **Support food taxes^a^** | |
| --- | --- | --- |
|  | OR | 95% CI |
| **FASI** |  |  |
| No Support  Ambivalence  High Support | Reference  1.380  1745 | 0.499-2.719  0.520-2.228 |
| **BMI** |  |  |
| Normal  Overweight  Obese | Reference  0.998  0.516** | 0.522-1.387  0.213-0.581 |

p<0.001**

FASI = Food addiction support index: No support (0-7); Ambivalence (8-12) and High support (13-20)

BMI = Body mass index: normal weight 18.5-24.9; overweight 25-29.9; obese > 30

^a^ Support for food taxes was generated by summing the scores for the two questions – 1) Food taxation would decrease obesity and 2) Food taxation is helpful – to develop an overall score from 0 to 8 (where strongly disagree = 0 to strongly agree = 4). These scores were then categorised as Support (Agree) and No Support (Ambivalent or Disagree).
