# Supplementary material for: Public Views on Food Addiction and Obesity: Implications for Policy and Treatment
Source: PLoS One. 2013 Sep 25;8(9):e74836. doi: 10.1371/journal.pone.0074836 (PMC3783484; doi:10.1371/journal.pone.0074836)
Supplement: Table S9 — Demographic characteristics of sample based upon Food Addiction Support Index (FASI). (DOCX) [file pone.0074836.s009.docx]

Table S9. Demographic characteristics of sample based upon Food Addiction Support Index (FASI).

| **Sample Characteristics** | No support  (n=39)  n (%) | Ambivalence  (n=78)  n (%) | High support (n=362)  n (%) |
| --- | --- | --- | --- |
| **Sex** | | | |
| *Female* | 21 (5) | 57 (15) | 305 (80) |
| *Male* | 17 (18) | 20 (22) | 56 (60) |
| **Age** | | | |
| *18-34* | 12 (5) | 40 (18) | 175 (77) |
| *35+* | 27 (11) | 38 (15) | 187 (74) |
| **BMI** | | | |
| *Normal weight* | 17 (7) | 52 (23) | 159 (70) |
| *Overweight* | 9 (9) | 16 (15) | 79 (76) |
| *Obesity* | 12 (9) | 9 (7) | 112 (84) |
| **Education** | | | |
| *Non-University* | 4 (4) | 17 (16) | 88 (81) |
| *College graduate* | 15 (9) | 17 (10) | 134 (81) |
| *Postgraduate degree* | 20 (10) | 44 (22) | 140 (69) |
| **Country of Residence** | | | |
| *United States* | 23 (11) | 35 (16) | 157 (73) |
| *Australia* | 16 (6) | 43 (16) | 205 (78) |

FASI = Food addiction support index: No support (0-7); Ambivalence (8-12) and High support (13-20)

BMI = Body mass index: normal weight 18.5-24.9; overweight 25-29.9; obese > 30
